# Genomic characterization and evolutionary analysis of a Getah virus variant from piglets in central China

**DOI:** 10.3389/fmicb.2025.1515632

**Published:** 2025-02-05

**Authors:** Zhenhua Guo, Yao Jiang, Peng Li, Gaiping Zhang

**Affiliations:** ^1^Key Laboratory of Animal Immunology of the Ministry of Agriculture, Institute for Animal Health, Henan Academy of Agricultural Sciences, Zhengzhou, China; ^2^College of Veterinary Medicine, Northwest A&F University, Yangling, China; ^3^Department of Veterinary Diagnostic and Production Animal Medicine, Iowa State University, Ames, IA, United States; ^4^Longhu Laboratory, Zhengzhou, China; ^5^Jiangsu Co-Innovation Center for Prevention and Control of Important Animal Infectious Diseases and Zoonoses, Yangzhou, China

**Keywords:** Getah virus, genomic characteristics, genetic evolution, E2 protein, positive selection

## Abstract

**Introduction:**

As a re-emerging mosquito-borne virus, Getah virus (GETV) has been found in more than 10 countries surrounded by the Pacific Ocean and shows a broadly host tropism, raising concerns for the potential risk to public health. Horses and pigs are susceptible to GETV and play pivotal roles to the GETV adaptive evolution.

**Methods:**

Here, we reported a GETV outbreak in a pig farm with 20% suckling piglets losses. The genomic characteristics and evolutionary relationship of the pathogenic viral strain were also analyzed.

**Results:**

The isolate GETV-HeN202309 shared the highest nucleotide identity of 99.8% with strains from Guangdong and Sichuan province, suggesting it is an imported transmission. Although the phylogenetic analysis divided GETV into four groups (groups I–IV), only strains in group III were dominant and widely circulating in the fields. Furthermore, several amino acid substitutions in E2 protein were identified among different GETV groups and the substitution at D262th N site led to an additional glycosylation modification. Besides, six amino acid sites were under positive selection of E2 protein. Most of these special sites were distributed in domain A, B, and C of E2 protein, which are usually associated with the GETV infection and immune response.

**Discussion:**

Our study expands knowledge of GETV pathogenicity and deepens understanding of GETV genetic and adaptive evolution, which would be valuable for the development of diagnosis and prevention for GETV.

## 1 Introduction

Getah virus (GETV), along with Chikungunya virus (CHIKV), O’nyong-nyong virus (ONNV), and Mayaro virus (MAYV), belongs to the genus *Alphavirus* within the *Togaviridae* family (Kim and Diamond, 2023; Zhai et al., 2024). As mosquito-borne viruses, most of them have been reported to cause severe and life-threatening infectious diseases in human and continue to pose a significant challenge to the public health (Kim and Diamond, 2023; Shi et al., 2022b). GETV was first isolated from *Culex gelidus* mosquitoes in Malaysia in 1955 (Nemoto et al., 2015), since when it has been reported in more than 12 countries and become an emerging mosquito-borne virus in the Eurasia and the Pan-Pacific region (Li et al., 2022; Shi et al., 2022b).

GETV is a lipid-enveloped, positive-sense single-stranded RNA virus (Li et al., 2017). The genome length is about 11,000 to 12,000 nucleotides (nt), which contains two open reading frames (ORFs). The first ORF account for two thirds of the viral genome and encodes four nonstructural proteins (nsp1–4), which are associated with viral RNA transcription, replication, polyprotein cleavage, and RNA capping. The second ORF mainly encodes viral structural proteins (C, E3, E2, 6K and E1) (Pan et al., 2023). Among them, E2 is the main functional protein that is responsible for viral entry into host cells during infection, causing disease and triggering host immune responses (Li et al., 2022; Wang N. et al., 2022).

More than 20 years after GETV was first found in mosquitoes in Malaysia, prevalence of GETV in horses was first reported in Japan in 1970s and in pigs in 1980s (Kuwata et al., 2018; Li et al., 2022). Subsequently, India, Thailand and China have also reported the infection of GETV in pigs and horses (Li et al., 2022). Recent studies also confirmed that GETV could infect cattle, blue foxes, and red pandas (Li et al., 2017; Shi et al., 2019; Shi et al., 2022b). More importantly, seroepidemiological analysis showed that specific antibodies against GETV have been identified in human sera in Malaysia, Australia and China (Li et al., 2022; Zhao et al., 2023). The expansion of GETV’s host range and geographical distribution has raised concerns from scientists. The virus is considered to pose a potential challenge to the public health.

Here, we reported a case of GETV infection in suckling piglets. The outbreak had led to severe economic losses with about 20% mortality rate in piglets. The genomic homology, evolutionary relationships, and molecular characteristics of E2 protein were also analyzed in detail. Our studies are valuable to deepen our understanding on prevalence and pathogenicity of GETV.

## 2 Materials and methods

### 2.1 Case description and etiology investigation

In September 2023, a severe unknown disease broke out in the suckling piglet population on a breed-wean farm in Henan province, central China. The infected piglets initially showed fever, and died within 48 h. Tissues (lung and spleen) and serums were collected and sent to a third-party veterinary diagnostic laboratory (Sino-Science Gene Company, Luoyang, Henan) for molecular testing by real-time PCR, including the African swine fever virus (ASFV), porcine reproductive and respiratory virus (RRRSV), classical swine fever virus (CSFV), and porcine pseudorabies virus (PRV). Then, lung and spleen tissues were collected and delivered to Health Management Center of Boehringer Ingelheim Biopharmaceutical (China) Co., Ltd. for nanopore sequencing.

### 2.2 Nanopore sequencing

To explore the potential causative agents, a combination of DNA sequencing and transcriptome sequencing were conducted using the nanopore platform. Briefly, the mixed tissue specimens (lung and spleen) were homogenized, diluted in phosphate-buffered saline (PBS) and centrifuged at 3,000 rpm for 5 min. The DNA was extracted from supernatant using the MagMAX prepackaged viral RNA/DNA kit (Thermo Fisher Scientific). Simultaneously, the extraction of RNA was performed by using the QIAamp^®^ Viral RNA Mini kit (Qiagen) and RNA Clean & Concentrator-5 (DNase Included) kits (Zymo Research). To seek the potential viral RNA, we employed the sequence-independent single primer amplification (SISPA) method to prepare the cDNA, which operates by using random primers for amplification. Subsequently, the extracted and amplified products underwent purification using the Agencourt AMPure XP magnetic beads (Beckman Coulter). Then, the nucleic acids were prepared for sequencing by creating a genomic library. This library preparation was carried out using the Ligation Sequencing Kit (SQK-LSK110) and Native Barcoding Expansion 1–12 (NBD104) strictly following the manufacturer’s instructions (Oxford Nanopore Technologies). Finally, the genomic library was sequenced on the MinION Mk1C sequencer (Oxford Nanopore Technologies). The sequencing results from the MinION Mk1C sequencer were split into separate FASTQ format files for each sample, and species classification was performed using Kraken2 software (Lu et al., 2022). Additionally, for maintaining and reusing the flow cell, we utilized the ONT Flow Cell Wash Kit (EXP-WSH003) (Oxford Nanopore Technologies), ensuring optimal performance and cost-effectiveness in the sequencing experiments.

### 2.3 Determination of GETV genome sequence

According to the result of nanopore sequencing, we designed 12 primer pairs based on the reference strain GDHYLC23 (GenBank No. OR487192) (Supplementary Table S1). Lung and spleen were homogenized and centrifuged at 12,000 rpm for 5 min, 4°C. Then, total RNA was extracted from the supernatant of homogenate and reversely transcribed into cDNA. A conventional reverse transcriptive PCR was carried out to amplify the genome sequence. The amplicons were purified and sequenced using the Sanger method (Sangon Biotech Co., Ltd.). Finally, the complete genome sequence of GETV was assembled using the DNASTAR version 7.1 software.

### 2.4 Homological and phylogenetic analysis

The homology of isolate in this study with other reference strains was analyzed using the MegAlign program (DNASTAR version 7.1, Inc., Madison, WI, United States) and Simplot version 3.5.1 based on the complete genome and E2 sequence. To explore the evolutionary characterization, available sequences of the GETV strains (until June 30, 2024) were obtained from the GenBank (Supplementary Table S2). All the genome sequences firstly went through alignment using the Multiple Alignment using Fast Fourier Transform (MAFFT) software. Then, based on the alignment result, the phylogenetic tree was deduced using neighbor-joining method with MEGA 11.0 software (Tamura et al., 2021). The phylogenetic tree based on the *E2* gene was directly constructed by MEGA11.0.

### 2.5 Recombination and sequence alignment analysis

All the genome alignment sequences were scanned by recombination detection program 4 (RDP 4.0) software with seven methods including RDP, GENECONV, BootScan, Maxchi, Chimaera, Siscan and 3Seq (Martin et al., 2015). If more than six of the seven methods yield a *p*-value above the threshold of 0.05, the presence of recombinant events is assumed, which would be further verified by the Simplot 3.5.1 software. The sequence alignment of E2 protein was analyzed by clustal *W* method using the MegAlign program (DNASTAR version 7.1, Inc., Madison, WI, United States).

### 2.6 Amino acid sites selection and glycosylation analysis

The positive amino acid sites selection on E2 protein was inferred using the Datamonkey^1^ with methods of Mixed Effects Model of Evolution (MEME), Fast Unconstrained Bayesian App Roximation for inferring selection (FUBAR), and Single Likelihood Ancestor Counting (SLAC). Positive selection was determined with a *p*-value cut-off of 0.1 for MEME and SLAC, and 0.9 for FUBAR. N-glycosylation sites in the E2 amino acid sequence were predicted by the NetNGlyc-1.0 server.^2^ The E2 protein models were created using the cryo-electron microscopy structure of GETV (PDB ID. 7VGA) with PyMol 2.5 software (Molecular Graphics System, Version 2.0 Schrödinger, LLC. https://pymol.org). The distance measurement was completed with the wizard tool of the program.

## 3 Results

### 3.1 Clinical status and laboratory diagnosis

In September 2023, a severe disease occurred in the farrowing room of a pig farm in Henan province, central China. The pig farm owned 286 sows, all of which were purchased at one time from a commercial breeding company. The infected piglets began exhibiting clinical signs around 7 days of age, with 35.2% (32/91) of litters cumulatively affected. However, the farrowing sows are clinically healthy. The infected piglets showed fever (>40°C), depression, unable to stand, and vomiting milk, followed by ataxia and tremors (Supplementary Video S1), and normally died in 48 h. Finally, more than 200 piglets were dead or humanely euthanized, accounting for a loss rate of about 20%.

**Figure 1:**
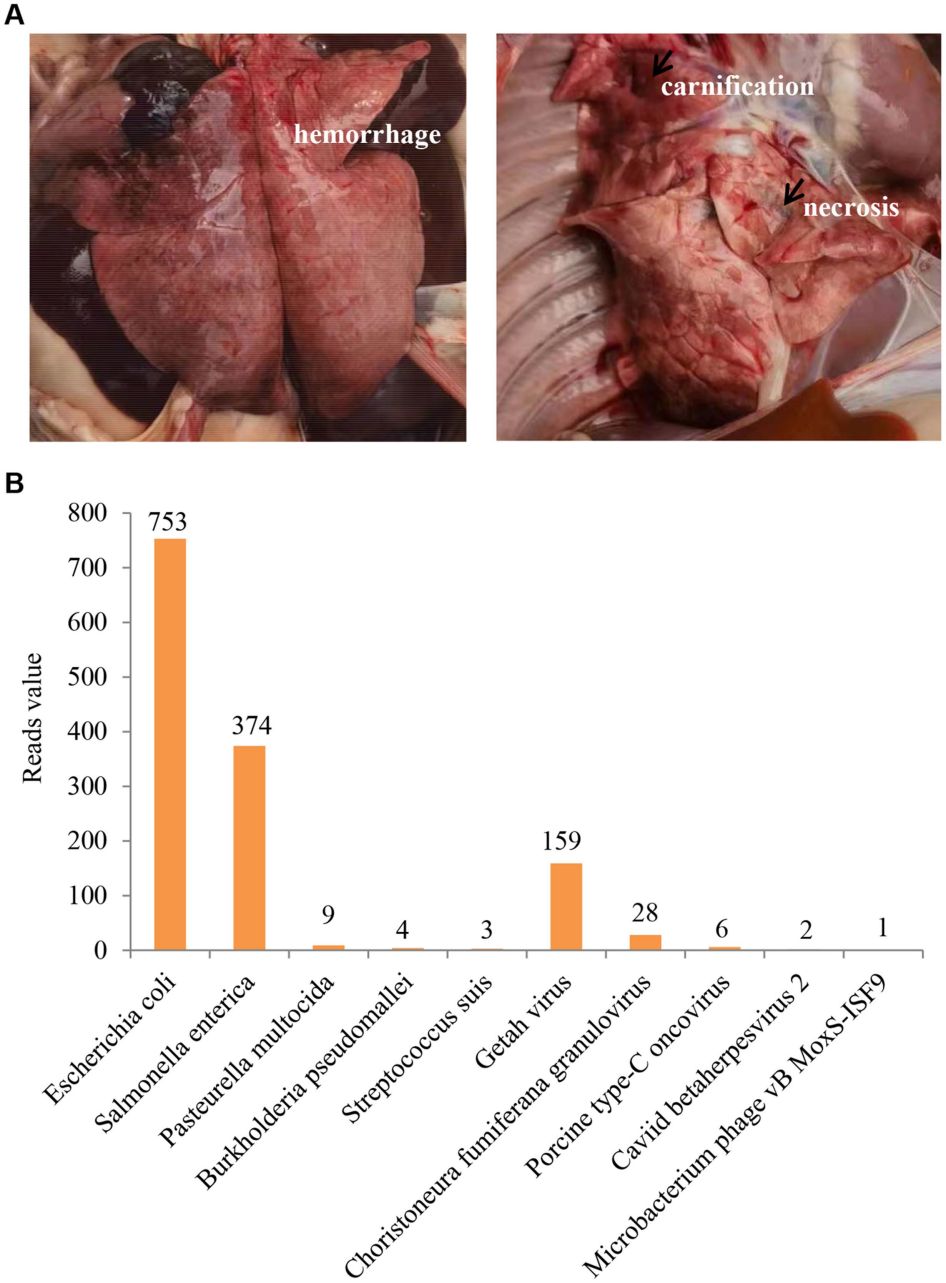
Clinical status and laboratory diagnosis. **(A)** Hemorrhage, carnification, and necrosis were observed in the lungs. **(B)** The result of nanopore sequencing. GETV has the highest reads value in viral pathogens.

Originally, the veterinary technicians at the pig farm conducted autopsies on five piglets with typical clinical symptoms and collected the samples (lung, spleen, and serum) for molecular test. Necropsy showed that hemorrhage, carnification, and necrosis were often seen in the lungs (Figure 1A). Needlepoint-like haemorrhage in the kidney and serrated edge from the spleen were also observed. The nucleic acid testing by the veterinary diagnostic laboratory excluded the infection of ASFV, PRRSV, CSFV, and PRV. After that, the veterinary technicians reached out to us for assistance. To identify the potential etiology, we further collected the lung and spleen tissues from two piglets to perform the nanopore sequencing with a combination of DNA sequencing and transcriptome sequencing technology by Health Management Center of Boehringer Ingelheim Biopharmaceutical (China) Co., Ltd. As shown in Figure 1B, GETV has the highest read value for the viral pathogens, while *Escherichia coli* and *Salmonella enterica* have a high reading value in the bacterial pathogens. According to the differences in pathogenicity of the pathogens, we speculated that GETV was the primary causative agent, and *Escherichia coli* and *Salmonella enterica* might be secondary infections.

### 3.2 Genome sequencing and homology analysis

**Figure 2:**
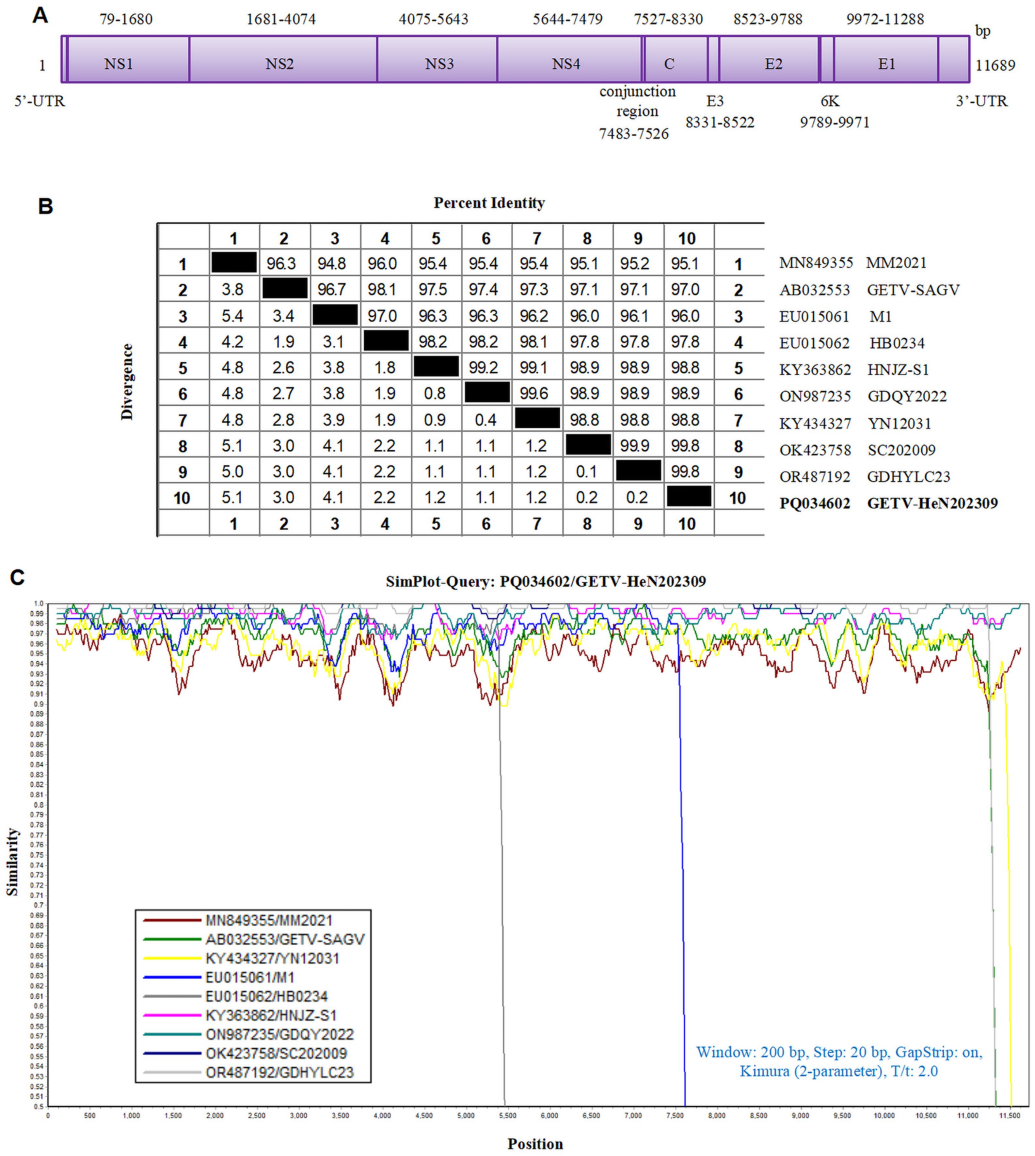
Homological analysis of the GETV isolate in this study. **(A)** The genomic schematic diagram of GETV-HeN202309 strain. **(B)** Homology analysis between the isolate GETV-HeN202309 and reference strains based on the complete genome. **(C)** Sequence similarity was analyzed between GETV-HeN202309 and representative strains.

In recent years, GETV infection has been reported in pigs in several provinces in China and is a re-emerging viral disease that requires greater attention (Lu et al., 2020). To investigate the genetic characteristic of GETV strain in this study, we sequenced the complete genome and submitted to GenBank with an accession number PQ034602, named GETV-HeN202309. The genome size is 11, 689 nucleotide (nt), including a short 3′ UTR (78 nt) and long 5′ UTR (401 nt). Two large ORFs, linked by a non-coding connecting regions (44 nt), encode non-structural proteins (7,404 nt, further cleaved into NS1, NS2, NS3, and NS4) and structural proteins (3,762 nt, further cleaved into C, E3, E2, 6K, and E1) (Figure 2A).

The genomic sequence alignment with the representative strains showed that the GETV-HeN202309 displayed the highest nucleotide identity of 99.8% with GDHYLC23 (GenBank No. OR487192) and SC202009 (GenBank No. OK423758) strain, which were isolated in Guangdong province (south of China) in 2023 and Sichuan province (southwest of China) in 2020, respectively. Furthermore, GETV-HeN202309 shared 95.1% nucleotide identity to prototype strain MM2021 (GenBank No. 849355) isolated in 1955 in Malaysia and 97.0% with the earliest strain M1 (GenBank No. EF011023) isolated in 1964 in China (Figures 2B,C).

### 3.3 Phylogenetic analysis of GETVs

**Figure 3:**
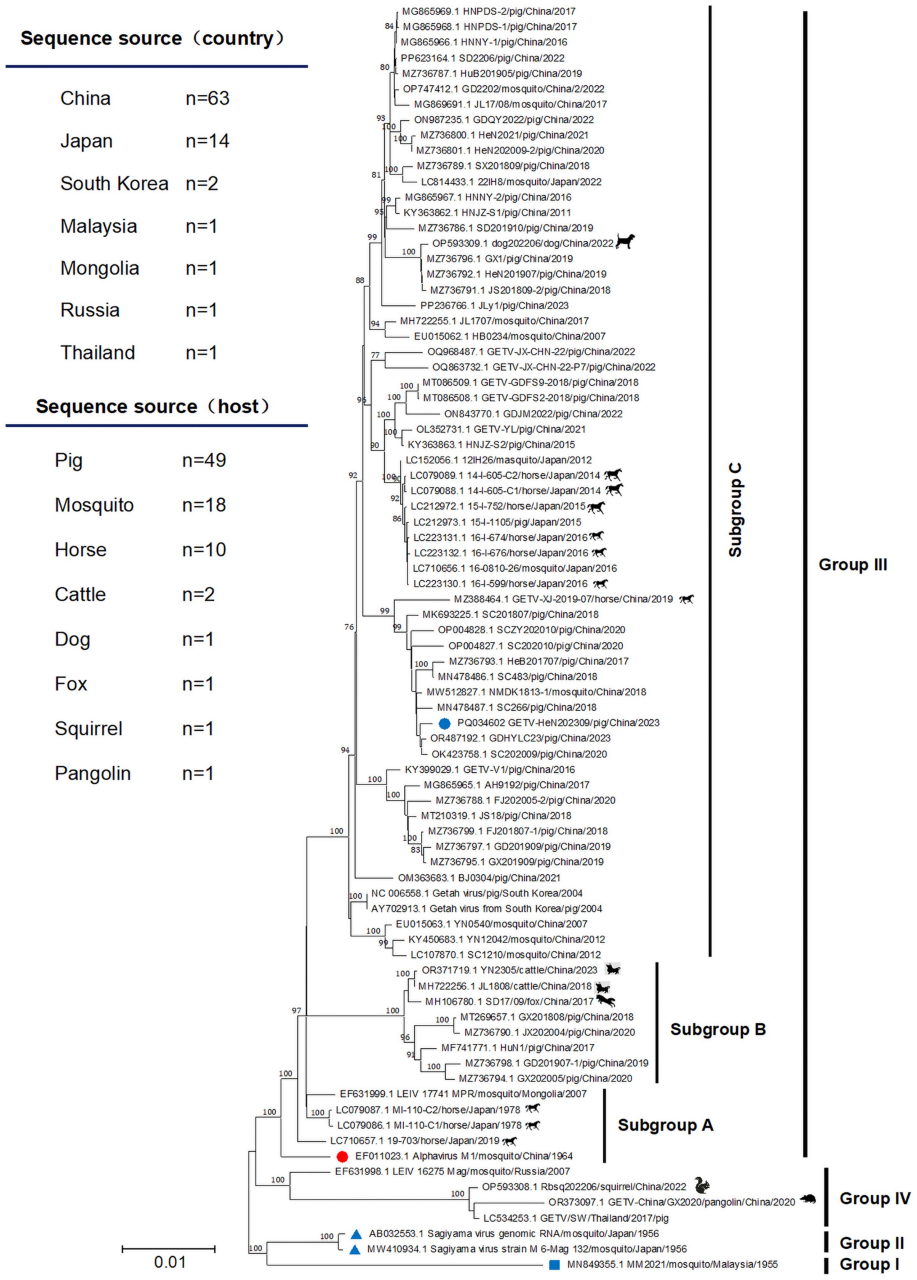
The phylogenetic tree was constructed based on the complete genome. The isolate GETV-HeN202309 and other 82 GETV genomic sequences available in GenBank (until July 31, 2024) were collected to perform nucleotide alignment by MAFFT software. Then, the phylogenetic tree were constructed in MEGA11 using the neighbor-joining method with the Kimura-2-parameter nucleotide substitution model. The blue square indicates the prototype strain isolated in Malaysia in 1955. The red circle displays the earliest isolated strain M1 in China in 1964. The blue triangle shows the only two group II strains in Japan in 1956. The blue circle indicates the isolate GETV-HeN202309 in this study. The geographic and host origins of the sequences are also shown at the top left of the figure.

**Figure 4:**
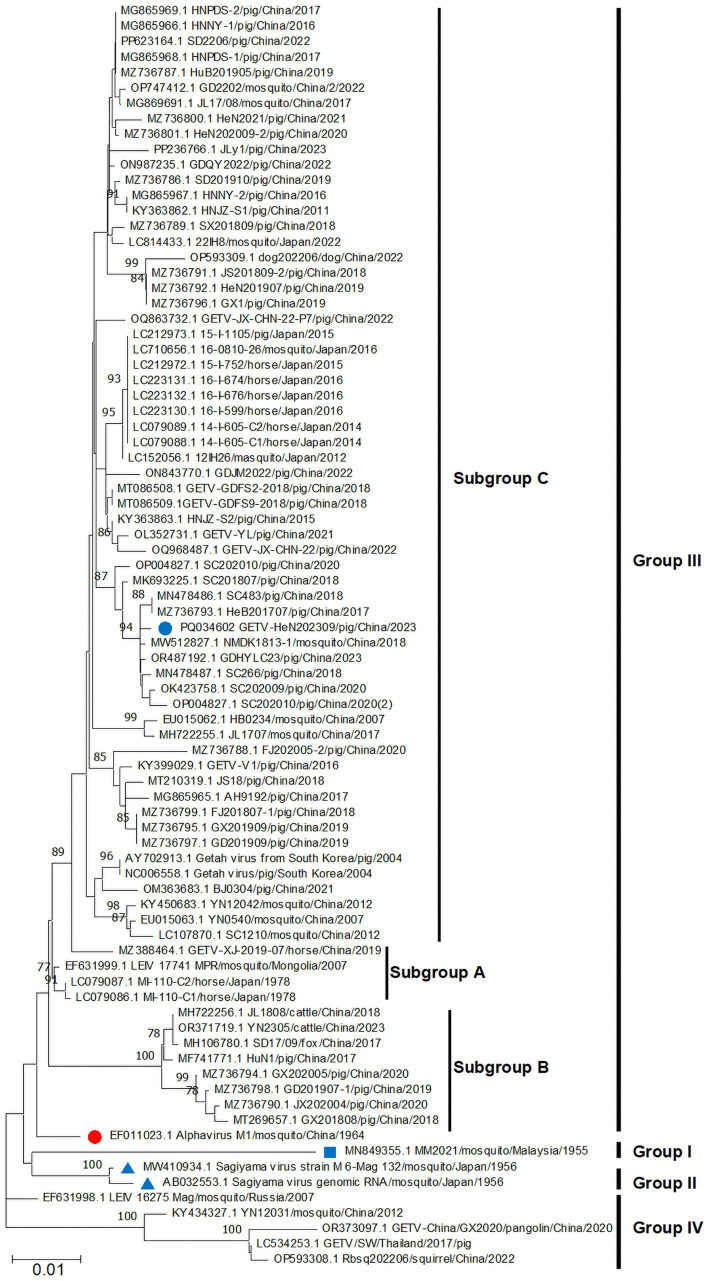
Phylogenetic analysis based on E2 nucleotide sequences of GETVs. The phylogenetic tree was inferred using MEGA 11.0 with the neighbor-joining method. The annotations were similar to Figure 3.

To explore the evolutionary relationships of GETV-HeN202309, we collected a total of 82 GETV genomic sequences available in GenBank (until July 31, 2024). Among the sequences, 75.9% (63/83) were from China, 16.9% (14/83) were from Japan. In terms of isolated hosts, 59.0% (49/83) were isolated from pigs, 21.7% (18/83) from mosquitoes, 12.0% (10/83) from horses, and remaining from animals included cattle, dog, foxes, squirrel, and pangolin (Figure 3). Furthermore, phylogenetic analysis was inferred based on the genome and E2 sequence (Figures 3, 4). The evolutionary tree showed that all the GETV strains could be divided into group I (prototype strain MM2021), group II (only reported in Japan in 1956), group III, and group IV. In total, 91.6% reported sequences belonged to group III, which was isolated from multiple hosts and prevalent across the Asia-Pacific region, and could be further divided into subgroup A, subgroup B, and subgroup C. The strain GETV-HeN202309 was clustered into subgroup C in group III. Interestingly, the sequences clustered together with GETV-HeN202309 were mainly from Sichuan (southwest of China), Guangdong (south of China), Inner Mongolia (north of China), and Xinjiang (northwest of China), but no viral sequences isolated from Henan province (central of China), suggesting that the strain GETV-HeN202309 may have been imported from other provinces to Henan, and the viral strains in this branch have a wider geographical distribution.

### 3.4 Recombination analysis

To evaluate whether recombinant events occurred among GETVs, all the 83 GETV genomic sequences were went through alignment by MAFFT firstly. Then, the recombinant analysis was performed by RDP 4.0 software with seven methods including RDP, GENECONV, BootScan, Maxchi, Chimaera, Siscan and 3Seq. Overall, no recombination event was detected within GETV strains.

### 3.5 Molecular characterization of E2 protein

**Figure 5:**
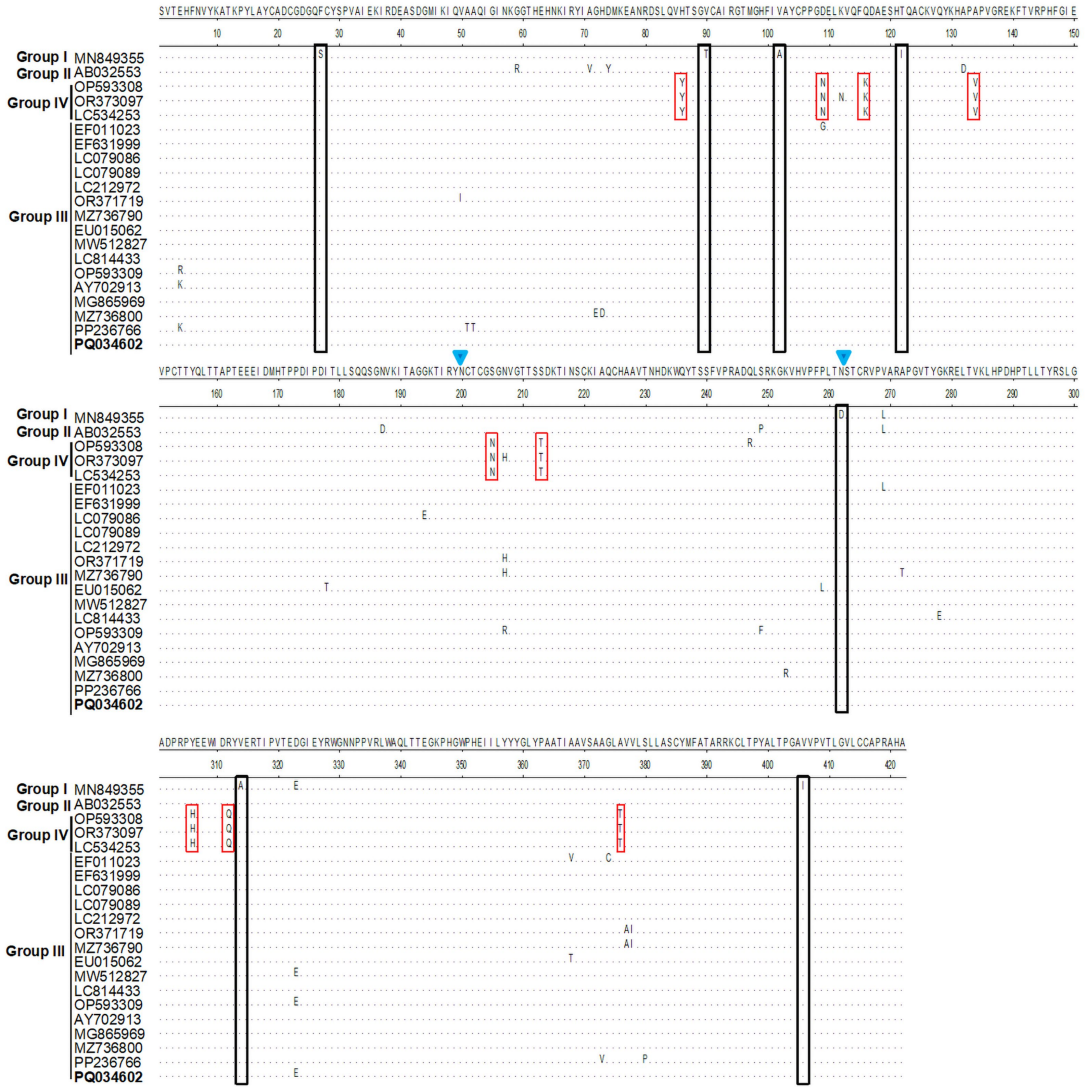
Residues substitution and glycosylation analysis for E2 protein. The E2 protein sequences were aligned using the MegAlign program. The unique amino acid mutations were showed in black or red squares. The glycosylation sites were indicated with blue inverted triangle.

The glycoprotein E2 protein of Alphaviruses plays key roles in attachment to cellular receptors (Kim and Diamond, 2023; Wang A. et al., 2022). To map the E2 molecular characteristics, we perform bioinformatics analysis using specific softwares. Homological analysis showed that the E2 sequences in group III shared 96.2–100% (nt)/97.6–100% (aa) identities with each other and 94.0–95.5% (nt)/96.7–98.1% (aa) similarities with the prototype strain MM2021 (group I). Furthermore, group IV showed the lowest similarity to other three groups, with nt homology being 92.1–93.0% to group I, 93.9–94.7% to group II, and 93.9–96.0% to group III, respectively (Table 1). The E2 protein sequence alignment showed that several aa mutations were observed among the different GETV strains. Briefly, compared to the prototype strain MM2021, seven aa substitutions (S27th F, T90th V, A102th V, I122th T, D262th N, A314th V, and I406th V) were found in groups II–IV. Specifically, the E2 protein in group IV shared nine unique amino acids (86th Y, 109th N, 116th K, 134th V, 205th N, 213th N, 307th H, 312th Q, and 376th T) (Figure 5). Considering the important role of glycoproteins in the viral life cycle and immune evasion, we further evaluated the N-glycosylation sites on E2 protein. As shown in Figure 5, two N-glycosylation sites (N200 and N262) were identified. Compared with the prototype strain MM2021, the other three groups shared an additionally glycosylated site at the 262th.

**Table 1:** Homological analysis of E2 gene among GETV strains.

nt/aa	Group I	Group II	Group III	Group IV
Group I	100%	94.9%/96.7%	94.0–95.5%/96.7–98.1%	92.1–93.0%/95.3–95.7%
Group II		99.6%/99.3%	95.8–97.9%/97.2–98.3%	93.9–94.7%/95.7–96.2%
Group III			96.2–100%/97.6–100%	93.9–96.0%/96.2–97.9%
Group IV				99.1–99.8%/99.3–99.8%

### 3.6 Amino acid selection analysis of E2 protein

**Figure 6:**
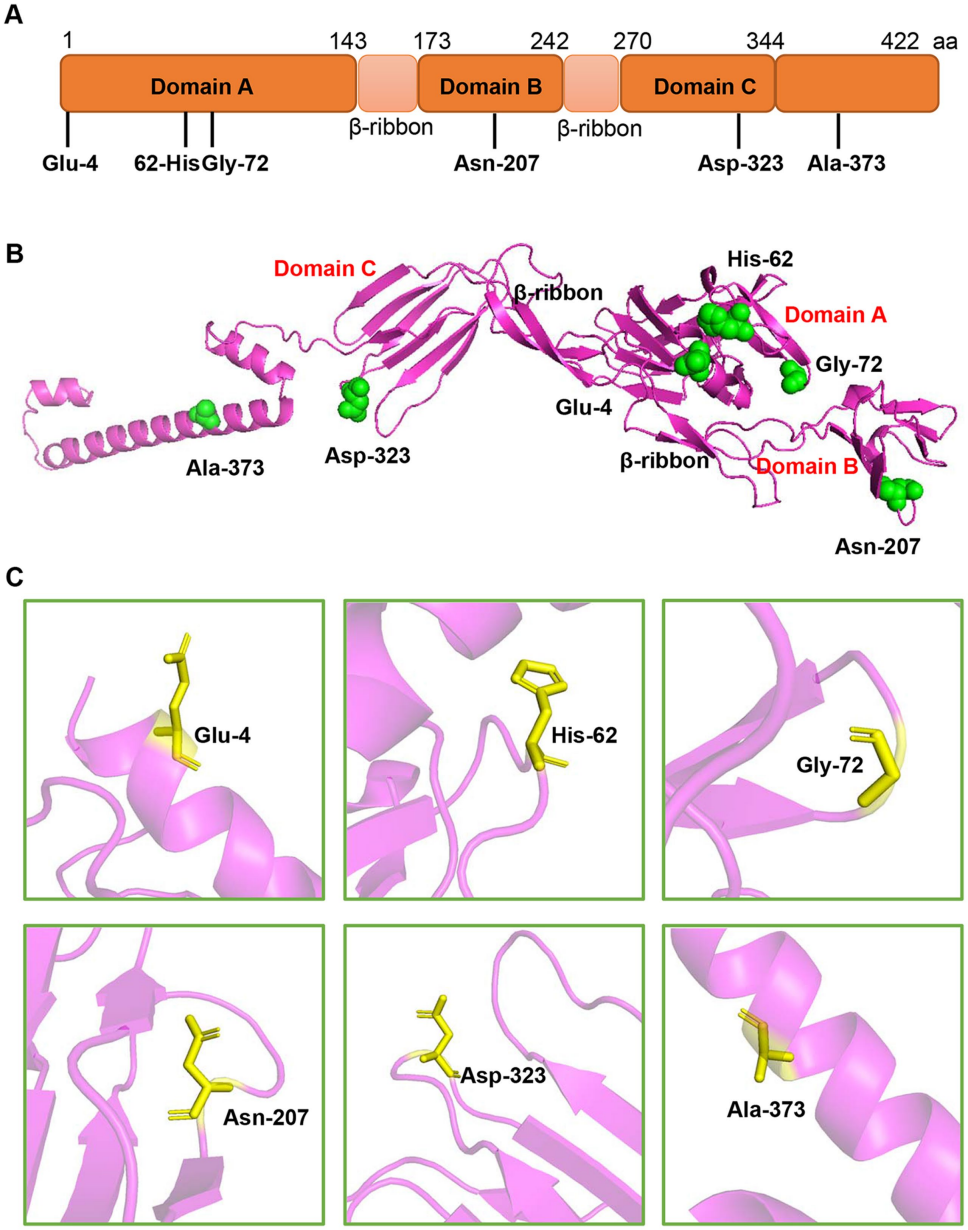
The location of the potential positive selection amino acid sites in E2 protein. **(A)** The selection sites were displayed in the schematic structure of E2 protein. E2 protein contains three domains-domain A (1–143aa), B (173–242aa), and C (270–344aa). **(B)** The selected sites (green spheres) were displayed on the structure of E2 monomer using PyMOL software. The residue sites (4, 62, and 72) are in the domain A. The site 207 is located in the domain B. The residue 323 is in the domain C and the residue 373 is in the C-terminus of E2 protein. **(C)** The single amino acid residue was zooming in the pink region and represented as sticks.

To better understand the adaptive evolution of GETV, we investigated the amino acids positive selection in E2 protein by the Datamonkey (see text footnote 1). Six aa sites (residues 4, 62, 72, 207, 323 and 373) were predicted to be under positive selection by the MEME method (Table 2). However, only two sites (residues 4 and 323) were identified by FUBAR method, and no positive sites were found by SLAC algorithm. The potential adaptive evolution sites were also displayed on a three-dimensional cartoon diagram using the cryo-electron microscopy (cryo-EM) structure of E2 protein (Figures 6A,B). Three sites (residues 4, 62, 72) were located in domain A that situated towards the centre of the trimeric spike. The 207th site was distributed in domain B, which is on the outermost tip of the spike. The 323th site was in domain C, which is proximal to the viral membrane. Detail of the residue location was also displayed in Figure 6C. Overall, selection pressure analysis revealed that the GETV E2 gene was under purifying selection.

**Table 2:** Amino acid selection analysis of the GETV E2 protein.

No.	Site	MEME (*p*-value)	FUBAR (post. pro)	SLAC (*p*-value)	Amino acid
1	4	0	0.993	0.137	Glu
2	62	0.09	0.671	0.514	His
3	72	0.08	0.213	0.889	Gly
4	207	0	0.59	0.668	Asn
5	323	0.07	0.976	0.275	Asp
6	373	0.08	0.849	0.296	Ala

## 4 Discussion

Over the past 20 years, GETV has expanded its regional distribution from low-latitude tropical regions to 60°north latitude and was endemic in more than 13 countries (Li et al., 2017). In addition to its natural host mosquitoes, GETV has been found to infect several animals, including horses, pigs, and cattle (Li et al., 2017; Shi et al., 2022b). Its economic losses to the farming industry remain unknown and the disease burden is severely underestimated. In China, the first GETV strain M1 was isolated from a *Culex* species collected in Hainan Island in 1964 (Gao et al., 2023). Currently, it has been found in more than 20 provinces (Lu et al., 2020). Seroepidemiological investigation showed that GETV has the highest positive rate in horses (~71.1%), followed by pigs (~51.1%) and cattle (~25.1%) (Liu et al., 2023; Shi et al., 2022a; Sun et al., 2022). More importantly, GETV neutralizing antibodies were also detected in chicken, ducks, and humans (Liu et al., 2023; Shi et al., 2022a; Sun et al., 2022). As an emerging mosquito-borne *Alphavirus*, GETV raised more concern and posed potential harm to animal safety and public health.

GETV infection could lead to fever, anorexia, depression, neurological symptoms (ataxia and tremors) in most of the GETV infected animals, and even death in foxes and piglets (Li et al., 2022; Nemoto et al., 2015). Generally, young animals often show more severe clinical signs. In addition to these shared clinical symptoms, rash and oedema of the limbs are often observed in horses (Nemoto et al., 2015). Here, we reported a GETV outbreak in about 7-day-old piglets, which caused high mortality with approximate 20% loss rate. However, all the farrowing sows (first parity) were clinical health. Necropsy showed gross lesions of carnification, hemorrhage, and necrosis in the lungs. This is different from previous findings that severe piglet death and sow reproductive disorders (abortion and abnormal estrus) were caused by a GETV variant GDHYLC23 infection (Chu et al., 2024). Homological analysis showed that the GETV-HeN202309 had the highest nucleotide identity of 99.8% with GDHYLC23 (GenBank No. OR487192), 95.1% identity with the prototype strain MM2021, and 97.0% identity with the earliest strain M1 in China, respectively. Interestingly, the GDHYLC23 strain contained a specific 32-nucleotide repeat insertion in the 3′untranslated region. However, the GETV-HeN202309 strain did not hold the unique insertion and only led to piglet losses in this case. Our data highlighted the diversity of clinical signs infected by different GETV strains.

Phylogenetic analysis based on whole genome sequence and E2 sequences showed that all the GETVs could be divided into four groups (groups I–IV), which were similar to previous studies (Li et al., 2017; Shi et al., 2022b). Group I was the prototype GETV strain (MM2021) isolated in Malaysia in 1955. Group II was reported in Japan in 1956 and only includes Sagiyama virus (SAGV) strain (GenBank No. MW410934 and AB032553). The strains in group IV were from Russia, China, and Thailand. Two specific genome sequences (GenBank No. OP593308 and OR373097) were from squirrel and pangolin, respectively, which implies that group IV might play an important role in the adaptive evolution of GETVs. Group III, represented by the first Chinese strain M1 isolated from mosquitoes in 1964, contains most of the GETV strains that is wildly circulating in the field. Strain GETV-HeN202309, isolated in this study, was clustered into subgroup C in group III. Interestingly, GETV-HeN202309 was more closely related to strains from Sichuan, Guangdong, and Hebei province. No other strains isolated from Henan province were found in the same clade. Thus, we speculated that the GETV-HeN202309 strain was imported into Henan from other provinces. It is also suggested that the epidemic status of GETV is complicated in China.

The high frequency of genetic variation is a significant feature of RNA viruses. Studies have estimated that the mean evolutionary rate of GETVs was about 3.41 × 10^−4^ substitutions/site/year (Zhao et al., 2023). Considering the E2 protein has key roles in attachment to cellular receptors and is a primary target of neutralizing antibodies (Kim and Diamond, 2023; Sun et al., 2022). We analyzed in detail the genetic variation and amino acids positive selection of E2 protein. Compared to the prototype MM2021, seven specific amino acid substitutions occurred in other GETV groups. Interestingly, the substitution at D262th N site led to an additional glycosylation modification. Previous study has shown that single amino acid substitution (arginine instead of lysine) at residue 253 of the E2 protein could lead to the attenuation of GETV (Wang N. et al., 2022). Considering that viral glycosylation plays important roles in viral pathobiology, it is essential to further explore the biological significance of the additional glycosylation at 253th site.

Furthermore, we identified six potential amino acid sites under positive selection of E2 protein. Most of these selective sites were distributed in domain A, B, and C of E2 protein, which usually have a vital role in the GETV infection and immune response (Kim and Diamond, 2023). Two sites Glu62 and His72 are localized in the residues 56–81 of domain A, the predominant region where both neutralizing and protective mAbs localized and host cell receptors interacted with. Domain B is another target of neutralizing and protective mAbs and is the most membrane-distal region of the *Alphavirus* E2-E1 heterodimer. Site Asn207 is in the domain B and located in a loop at the edge of the spike and exposed at the cell surface. The findings that epitopes for broadly neutralizing antibodies are located in the same region, suggesting residue 207 may have an effect on the attachment of GETV. Although domain C is much less accessible for mAbs to bind, study showed that two neutralizing mAbs against Venezuelan equine encephalitis virus (VEEV), mVEEV-19, and mVEEV-68 could bind residue 332 in domain C (Kim and Diamond, 2023; Wang N. et al., 2022). The positive selection site Asp323, exposed at the surface of the molecule near the membrane, was near this region. Considering GETV has a widely spectrum of host tropism, it will be invaluable to further deepen understand the adaptive evolution of GETV.

As a re-emerging mosquito-borne virus, GETV infection has been found in a variety of animals. GETV also has frequent cross-species transmission and can adapt to multiple hosts. The identification in multiple domestic animals highlights its transmission potential and public health risks. Importantly, its prevalence in China has increased significantly since 2016. It is imperative to conduct GETV monitoring and surveillance, especially cross-species infection to humans. Developing diagnostic tools and effective vaccines should also be a key priority.

## Data Availability

The datasets presented in this study can be found in online repositories. The names of the repository/repositories and accession number(s) can be found in the article/Supplementary material.

## Footnotes

^1^ http://www.datamonkey.org/

^2^ https://services.healthtech.dtu.dk/services/NetNGlyc-1.0/
