# Supplementary figures and images for: Genomic characterization and evolutionary analysis of a Getah virus variant from piglets in central China
Source: Front Microbiol. 2025 Feb 5;16:1515632. doi: 10.3389/fmicb.2025.1515632 (PMC11836007; doi:10.3389/fmicb.2025.1515632)

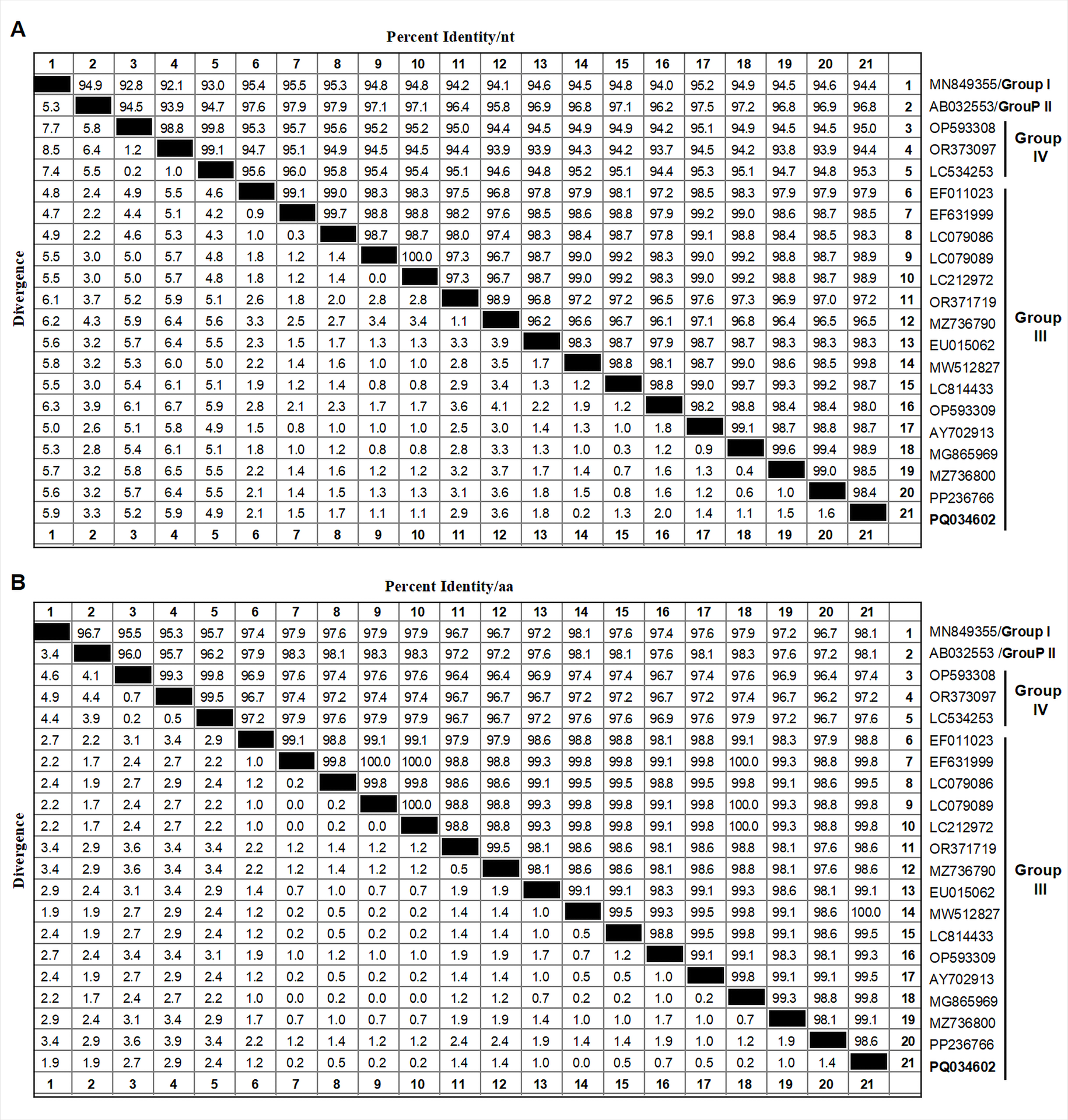

Supplement: Supplementary file 1 [file Image_1.TIF]
