# Supplementary material for: Genomic characterization and evolutionary analysis of a Getah virus variant from piglets in central China
Source: Front Microbiol. 2025 Feb 5;16:1515632. doi: 10.3389/fmicb.2025.1515632 (PMC11836007; doi:10.3389/fmicb.2025.1515632)
Supplement: Supplementary file 2 [file Table_1.DOCX]

**Table S1.** Primers used for genome sequence

| **Primer name** | **Sequence 5’-3’** | **position** |
| --- | --- | --- |
| P1-For | ATGGCGGACGTGTGACATC | 1 |
| P1-Rev | ATCCTCTGGTGTCACGTCAGTG | 1137 |
| P2-For | ACAGTCGATTACGCAGTTACCCAT | 973 |
| P2-Rev | TCGTGGAAAGGTGGATTGATTAG | 2168 |
| P3-For | ATAAATCGCAAGCTACACCACATAG | 1975 |
| P3-Rev | ATTTTGCCCAGCACACCTTAG | 3124 |
| P4-For | GTGGAAGACACTGTCGGGTGAC | 2946 |
| P4-Rev | TTTACGACCGCTTCCTCACTGT | 4136 |
| P5-For | GTTCCTGCTGTTTTCTAACTTTGAT | 3963 |
| P5-Rev | GGTCGTCTCCGATGGAAATGA | 5136 |
| P6-For | AGGTGAAGTGTGACCGAGTGCT | 4979 |
| P6-Rev | CGTGGTGCTTAGGGTAACATCT | 6175 |
| P7-For | TTTCTTAGAGGAGAATTATCCAACC | 6027 |
| P7-Rev | AGTACGGTGGTTTCTCACACATTG | 7159 |
| P8-For | CAACACGCTGCTGAACATTGTC | 6960 |
| P8-Rev | ATGGGTGTACTTTGAAGCGTCTG | 8087 |
| P9-For | TGCCTAGTCGGGGATAAAGTGAT | 7929 |
| P9-Rev | GCTGTGATCTTTACATTACCTGACTG | 9097 |
| P10-For | CCAGTAGGCAGAGAAAAATTCACC | 8925 |
| P10-Rev | GTTCCTCTCAATGTGAGCCTTATAC | 10037 |
| P11-For | GAAGAACCTGCTTTGCTGCTG | 9896 |
| P11-Rev | CCTGTATGGTGGCTACGTTCG | 10992 |
| P12-For | GCGTAATCAAGACCAACCCAGT | 10747 |
| P12-Rev | GGGGTAAAATATTAAAAAAACAAATTAG | 11721 |
