# Supplementary material for: Genomic characterization and evolutionary analysis of a Getah virus variant from piglets in central China
Source: Front Microbiol. 2025 Feb 5;16:1515632. doi: 10.3389/fmicb.2025.1515632 (PMC11836007; doi:10.3389/fmicb.2025.1515632)
Supplement: Supplementary file 3 [file Table_2.DOCX]

**Table S2.** The reference sequence information in this study**.**

| **No.** | **Strain name** | **Genbank no.** | **Country** | **Host** | **Collection year** |
| --- | --- | --- | --- | --- | --- |
| 1 | MM2021 | MN849355.1 | Malaysia | Mosquito | 1955 |
| 2 | Sagiyama virus strain M 6-Mag 132 | MW410934.1 | Japan | Mosquito | 1956 |
| 3 | Sagiyama virus genomic RNA/GETV-SAGV | AB032553.1 | Japan | Mosquito | 1956 |
| 4 | Alphavirus M1 | EF011023.1 | China | Mosquito | 1964 |
| 5 | YN2305 | OR371719.1 | China | Cattle | 2023 |
| 6 | JL1808 | MH722256.1 | China | Cattle | 2018 |
| 7 | SD17/09 | MH106780.1 | China | Fox | 2017 |
| 8 | HuN1 | MF741771.1 | China | Pig | 2017 |
| 9 | GX201808 | MT269657.1 | China | Pig | 2018 |
| 10 | JX202004 | MZ736790.1 | China | Pig | 2020 |
| 11 | GD201907-1 | MZ736798.1 | China | Pig | 2019 |
| 12 | GX202005 | MZ736794.1 | China | Pig | 2020 |
| 13 | GD2202 | OP747412.1 | China | Mosquito | 2022 |
| 14 | HNPDS-2 | MG865969.1 | China | Pig | 2017 |
| 15 | HNNY-1 | MG865966.1 | China | Pig | 2016 |
| 16 | SD2206 | PP623164.1 | China | Pig | 2022 |
| 17 | HNPDS-1 | MG865968.1 | China | Pig | 2017 |
| 18 | HuB201905 | MZ736787.1 | China | Pig | 2019 |
| 19 | JL17/08 | MG869691.1 | China | Mosquito | 2017 |
| 20 | GDQY2022 | ON987235.1 | China | Pig | 2022 |
| 21 | HeN2021 | MZ736800.1 | China | Pig | 2021 |
| 22 | HeN202009-2 | MZ736801.1 | China | Pig | 2020 |
| 23 | HNNY-2 | MG865967.1 | China | Pig | 2016 |
| 24 | HNJZ-S1 | KY363862.1 | China | Pig | 2011 |
| 25 | SX201809 | MZ736789.1 | China | Pig | 2018 |
| 26 | 22IH8 | LC814433.1 | China | Mosquito | 2022 |
| 27 | SD201910 | MZ736786.1 | China | Pig | 2019 |
| 28 | JL1707 | MH722255.1 | China | Mosquito | 2017 |
| 29 | HB0234 | EU015062.1 | China | Mosquito | 2007 |
| 30 | JLy1 | PP236766.1 | China | pig | 2023 |
| 31 | dog202206 | OP593309.1 | China | Dog | 2022 |
| 32 | HeN201907 | MZ736792.1 | China | Pig | 2019 |
| 33 | GX1 | MZ736796.1 | China | Pig | 2019 |
| 34 | JS201809-2 | MZ736791.1 | China | Pig | 2018 |
| 35 | GETV-JX-CHN-22 | OQ968487.1 | China | Pig | 2022 |
| 36 | 16-0810-26 | LC710656.1 | Japan | Mosquito | 2016 |
| 37 | 15-I-1105 | LC212973.1 | Japan | Pig | 2015 |
| 38 | 15-I-752 | LC212972.1 | Japan | Horse | 2015 |
| 39 | 16-I-674 | LC223131.1 | Japan | Horse | 2016 |
| 40 | 16-I-676 | LC223132.1 | Japan | Horse | 2016 |
| 41 | 12IH26 | LC152056.1 | Japan | Mosquito | 2012 |
| 42 | 16-I-599 | LC223130.1 | Japan | Horse | 2016 |
| 43 | 14-I-605-C2 | LC079089.1 | Japan | Horse | 2014 |
| 44 | 14-I-605-C1 | LC079088.1 | Japan | Horse | 2014 |
| 45 | GETV-GDFS9-2018 | MT086509.1 | China | Pig | 2018 |
| 46 | GETV-GDFS2-2018 | MT086508.1 | China | Pig | 2018 |
| 47 | GETV-YL | OL352731.1 | China | Pig | 2021 |
| 48 | HNJZ-S2 | KY363863.1 | China | Pig | 2015 |
| 49 | GDJM2022 | ON843770.1 | China | Pig | 2022 |
| 50 | YN12042 | KY450683.1 | China | Mosquito | 2012 |
| 51 | YN0540 | EU015063.1 | China | Mosquito | 2007 |
| 52 | SC1210 | LC107870.1 | China | Mosquito | 2012 |
| 53 | Getah virus | NC_006558.1 | South Korea | Pig | 2004 |
| 54 | Getah virus | AY702913.1 | South Korea | Pig | 2004 |
| 55 | BJ0304 | OM363683.1 | China | Pig | 2021 |
| 56 | AH9192 | MG865965.1 | China | Pig | 2017 |
| 57 | GD201909 | MZ736797.1 | China | Pig | 2019 |
| 58 | GX201909 | MZ736795.1 | China | Pig | 2019 |
| 59 | FJ201807-1 | MZ736799.1 | China | Pig | 2018 |
| 60 | JS18 | MT210319.1 | China | Pig | 2018 |
| 61 | FJ202005-2 | MZ736788.1 | China | Pig | 2020 |
| 62 | GETV-V1 | KY399029.1 | China | Pig | 2016 |
| 63 | GETV-JX-CHN-22-P7 | OQ863732.1 | China | Pig | 2022 |
| 64 | 19-703 | LC710657.1 | Japan | Horse | 2019 |
| 65 | LEIV 17741 MPR | EF631999.1 | Mongolia | Mosquito | 2007 |
| 66 | MI-110-C2 | LC079087.1 | Japan | Horse | 1978 |
| 67 | MI-110-C1 | LC079086.1 | Japan | Horse | 1978 |
| 68 | GETV-XJ-2019-07 | MZ388464.1 | China | Horse | 2019 |
| 69 | NMDK1813-1 | MW512827.1 | China | Mosquito | 2018 |
| 70 | GDHYLC23 | OR487192.1 | China | Pig | 2023 |
| 71 | SC202009 | OK423758.1 | China | Pig | 2020 |
| 72 | SC201807 | MK693225.1 | China | Pig | 2018 |
| 73 | SC266 | MN478487.1 | China | Pig | 2018 |
| 74 | HeB201707 | MZ736793.1 | China | Pig | 2017 |
| 75 | SC483 | MN478486.1 | China | Pig | 2018 |
| 76 | SCZY202010 | OP004828.1 | China | Pig | 2020 |
| 77 | SC202010 | OP004827.1 | China | Pig | 2020 |
| 78 | LEIV 16275 Mag | EF631998.1 | Russia | Mosquito | 2007 |
| 79 | GETV-China/GX2020 | OR373097.1 | China | Pangolin | 2020 |
| 80 | Rbsq202206 | OP593308.1 | China | Squirrel | 2022 |
| 81 | GETV/SW | LC534253.1 | Thailand | Pig | 2017 |
| 82 | YN12031 | KY434327.1 | China | Mosquito | 2012 |
| 83 | GETV-HeN202309 | PQ034602 | China | Pig | 2023 |
